# Supplementary figures and images for: Cross-protection against African swine fever virus upon intranasal vaccination is associated with an adaptive-innate immune crosstalk
Source: PLoS Pathog. 2022 Nov 9;18(11):e1010931. doi: 10.1371/journal.ppat.1010931 (PMC9645615; doi:10.1371/journal.ppat.1010931)

**S1 Fig.**


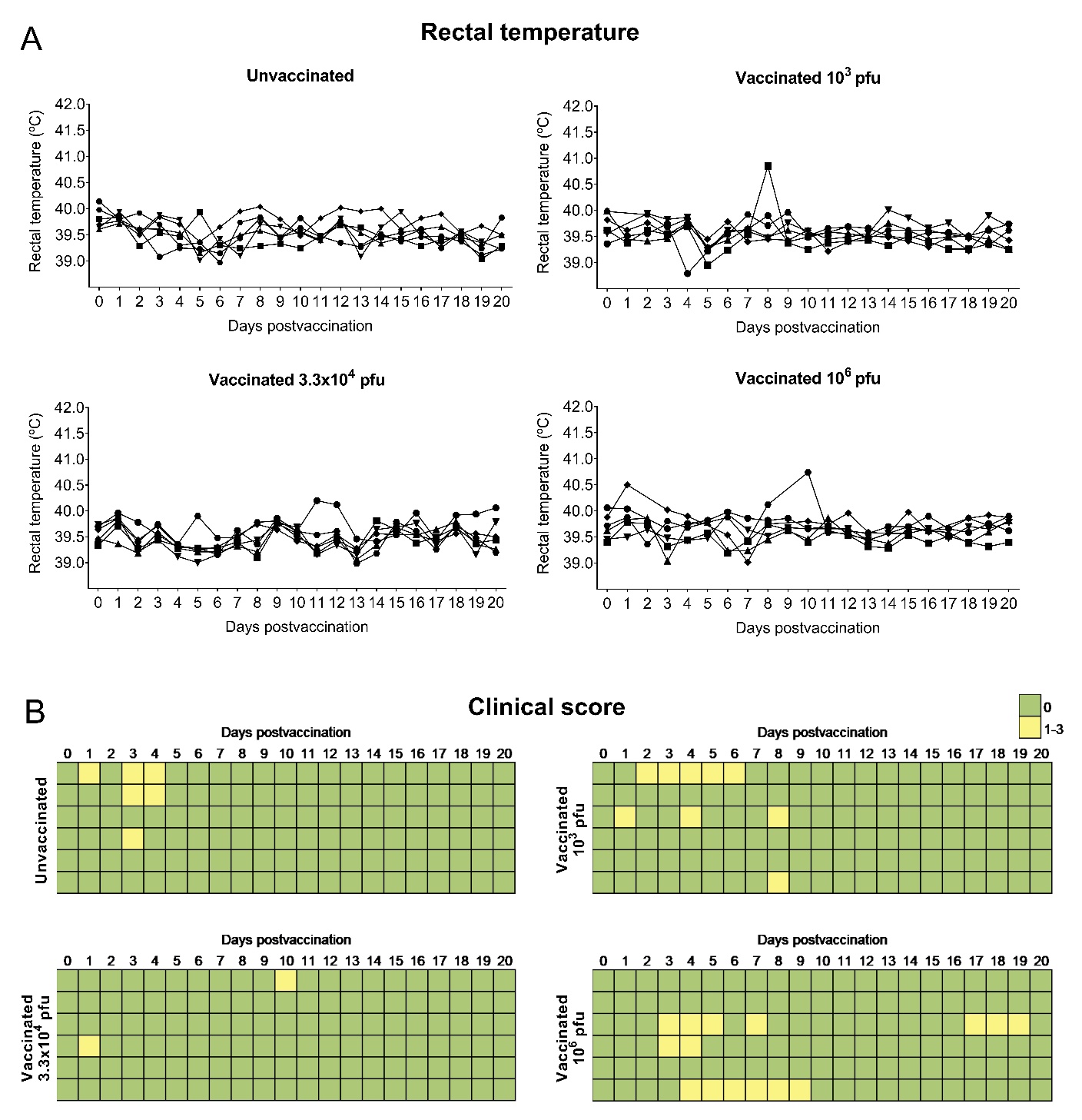

Supplement: S1 Fig — (A) Rectal temperatures from individual animals in each group. (B) Clinical scores measured throughout the experiment. Each row represents an animal within the group. (DOCX) [file ppat.1010931.s001.docx]

**S2 Fig.**


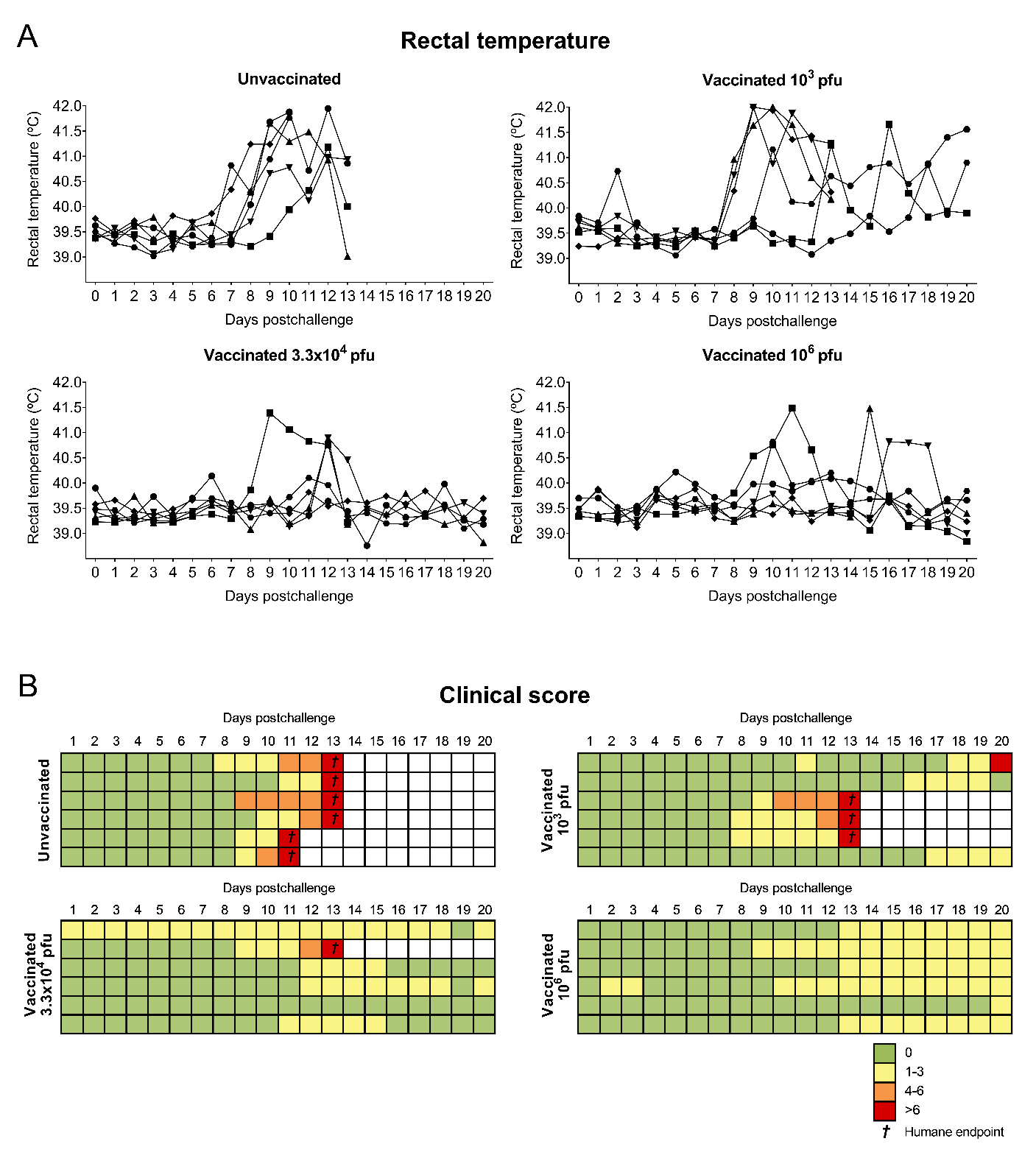

Supplement: S2 Fig — A) Rectal temperatures from individual animals in each group. (B) Clinical scores measured throughout the experiment. Each row represents an animal within the group. (DOCX) [file ppat.1010931.s002.docx]

**S3 Fig.**


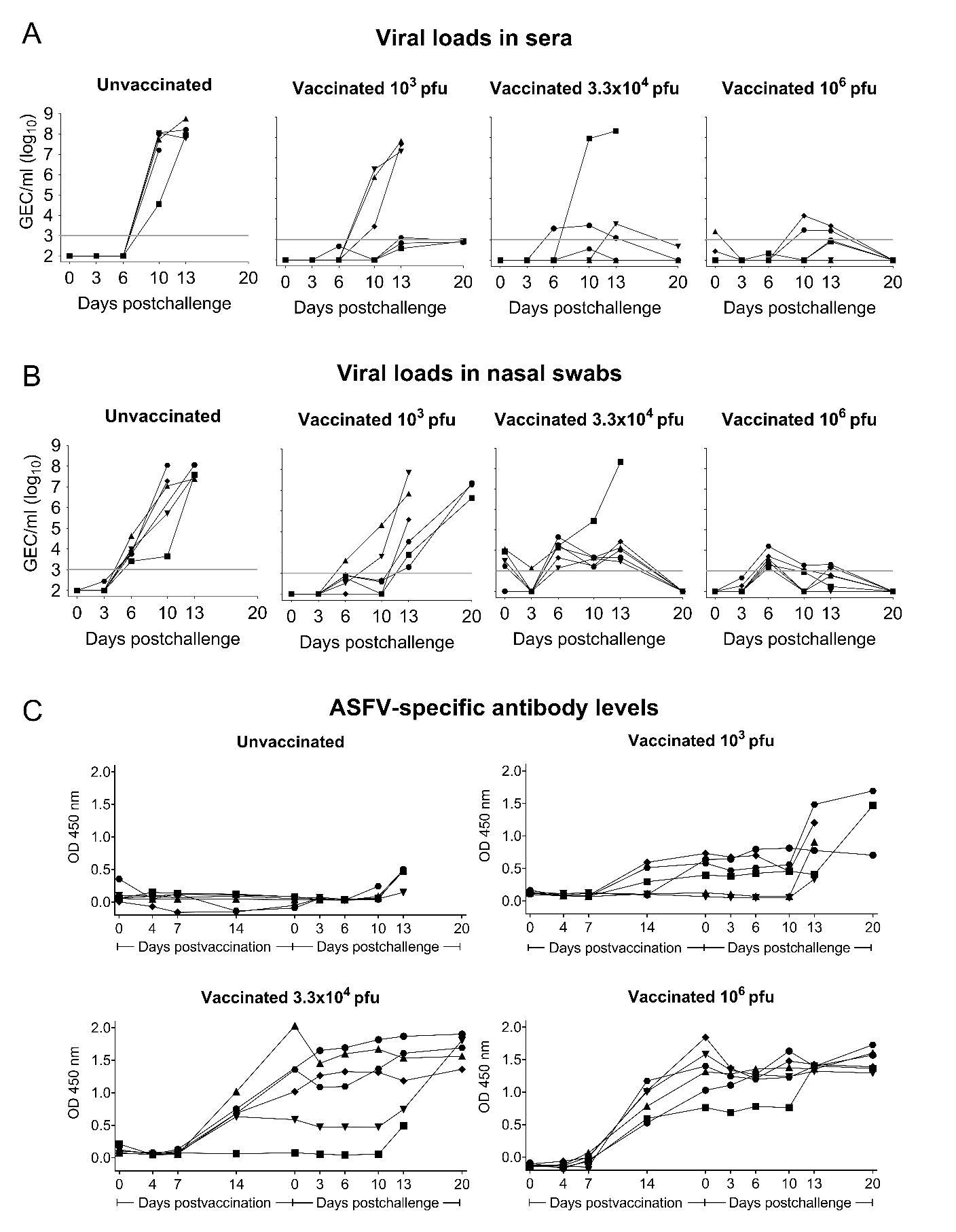

Supplement: S3 Fig — Virus titers in (A) sera and (B) nasal swabs measured by qPCR at the indicated time points after Georgia2007/1 direct-contact challenge. (C) ASFV-specific antibody levels in sera from vaccinated and unvaccinated pigs assessed by ELISA at the indicated time points. (DOCX) [file ppat.1010931.s003.docx]

**S4 Fig.**


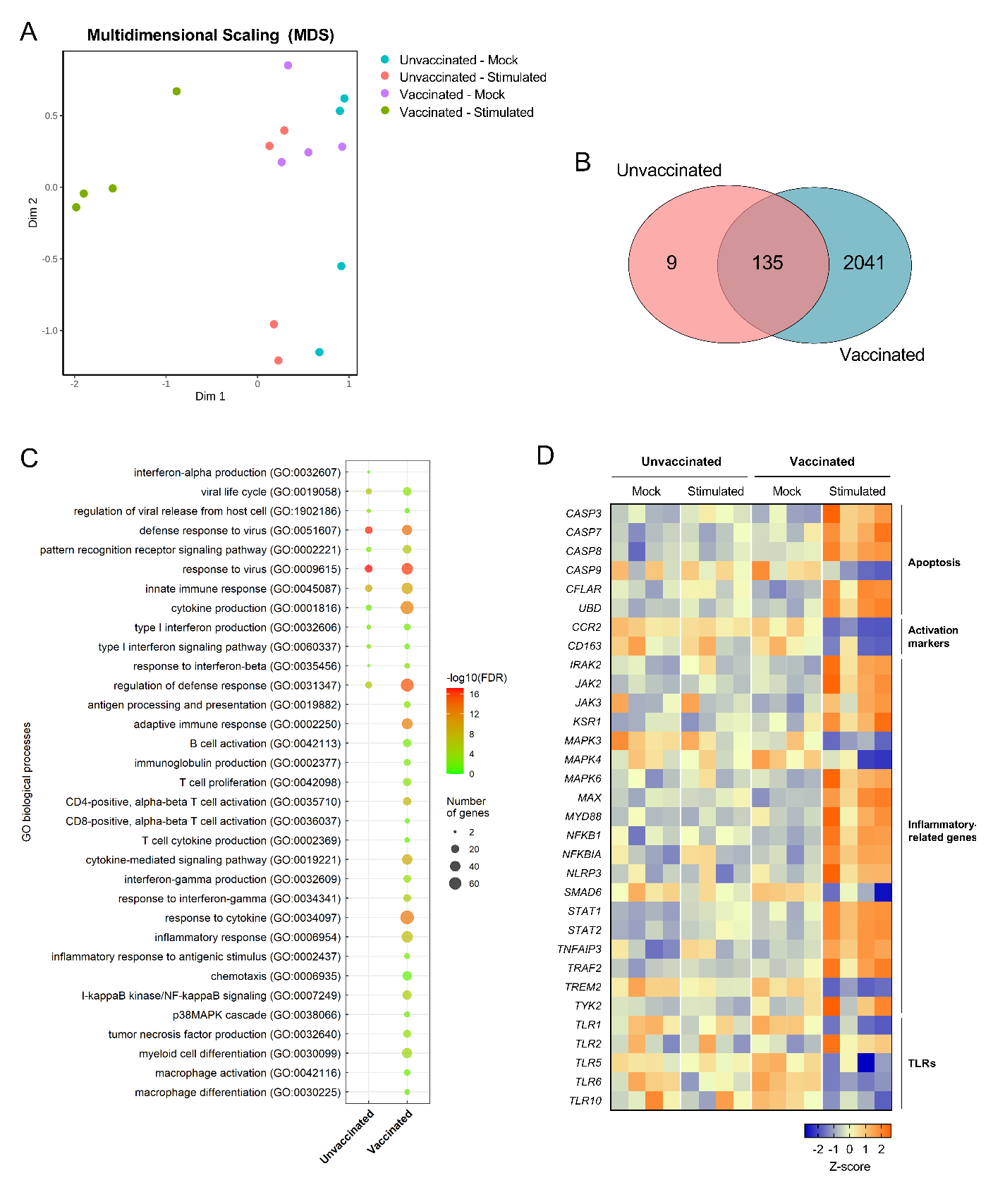

Supplement: S4 Fig — (A) Multidimensional scaling analysis of the normalized RNA-seq expression levels (log2CPM). (B) Venn diagram showing the number of overlapping and unique DE genes identified in the unvaccinated and vaccinated groups. (C) List of representative GO terms enriched in DE genes from BA71ΔCD2-stimulated PBMC from unvaccinated and vaccinated pigs. The size of the dots represents the number of DE genes associated with the GO term, and the color indicates the negative log10 value of the false discovery rate (FDR). (D) Heatmap depicting normalized RNA-seq-derived log2CPM values of representative DE genes (extension of Fig 2C). (DOCX) [file ppat.1010931.s004.docx]

**S5 Fig.**


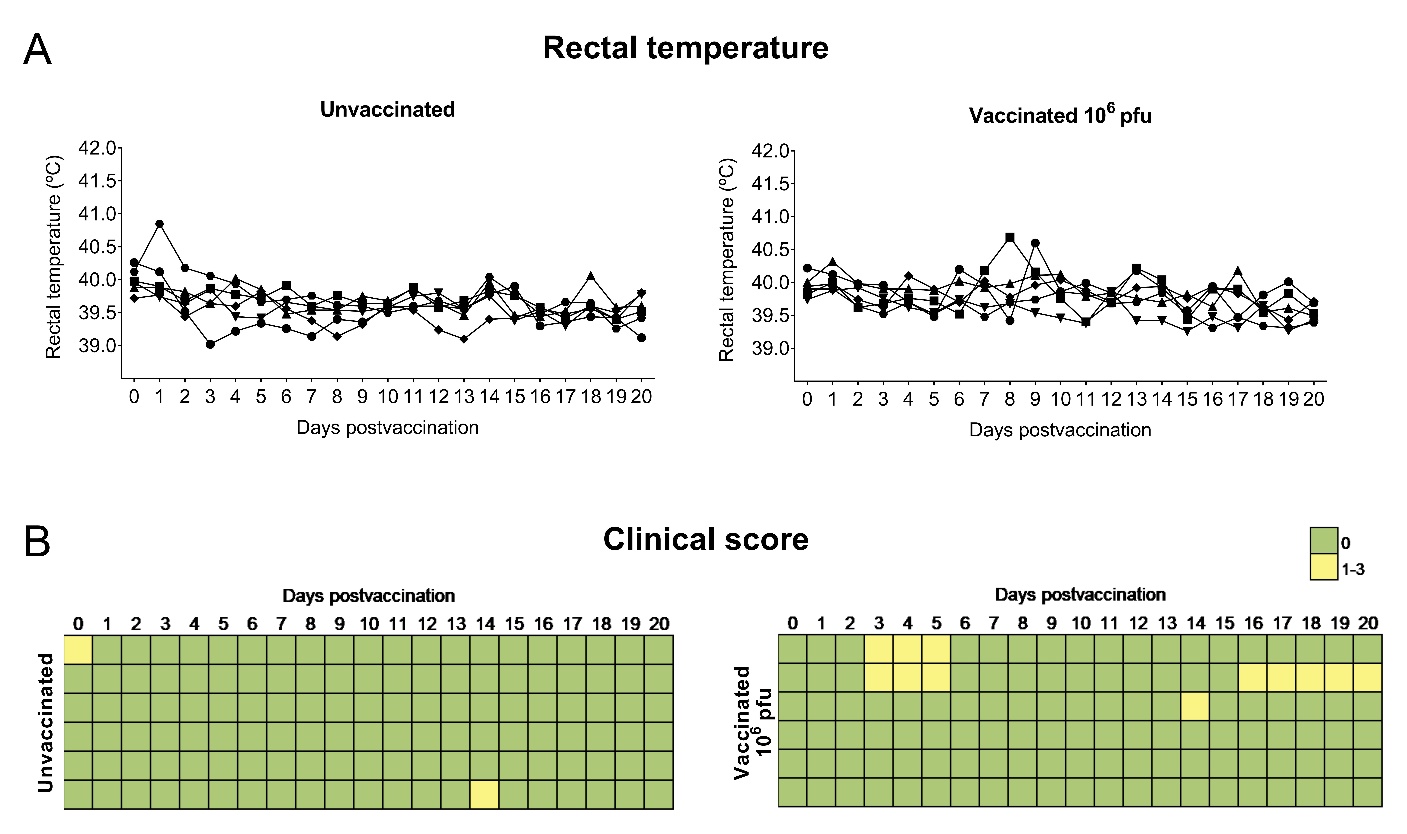

Supplement: S5 Fig — (A) Rectal temperatures from individual animals in each group. (B) Clinical scores measured throughout the experiment. Each row represents an animal within the group. (DOCX) [file ppat.1010931.s005.docx]

**S6 Fig.**


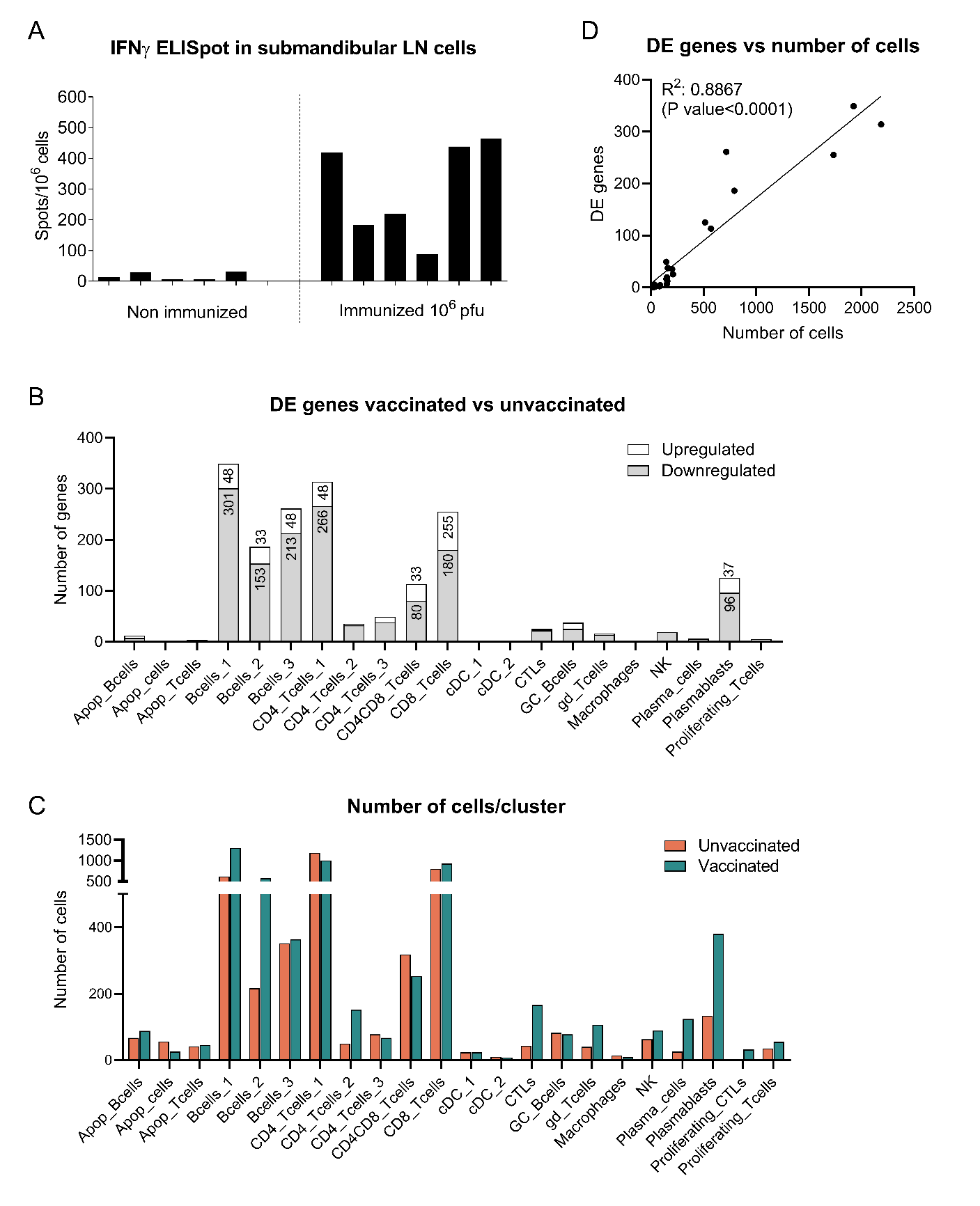

Supplement: S6 Fig — (A) Pigs were vaccinated with 106 pfu of BA71ΔCD2 (n = 6) and three weeks later levels of ASFV-specific cells in submandibular LN were measured by IFNγ ELISpot using BA71ΔCD2 as stimulus. Unvaccinated pigs (n = 6) were used as negative control. (B) Number of genes differentially expressed between the unvaccinated and the vaccinated pig in each cluster. (C) Number of cells in each cluster identified by scRNA-seq. (D) Pearson’s correlation between the number of DE genes and the number of cells in each cluster. Each dot represents a cluster, and the value of the total number of cells is the result of the addition of cells from each sample. (DOCX) [file ppat.1010931.s006.docx]

**S7 Fig.**


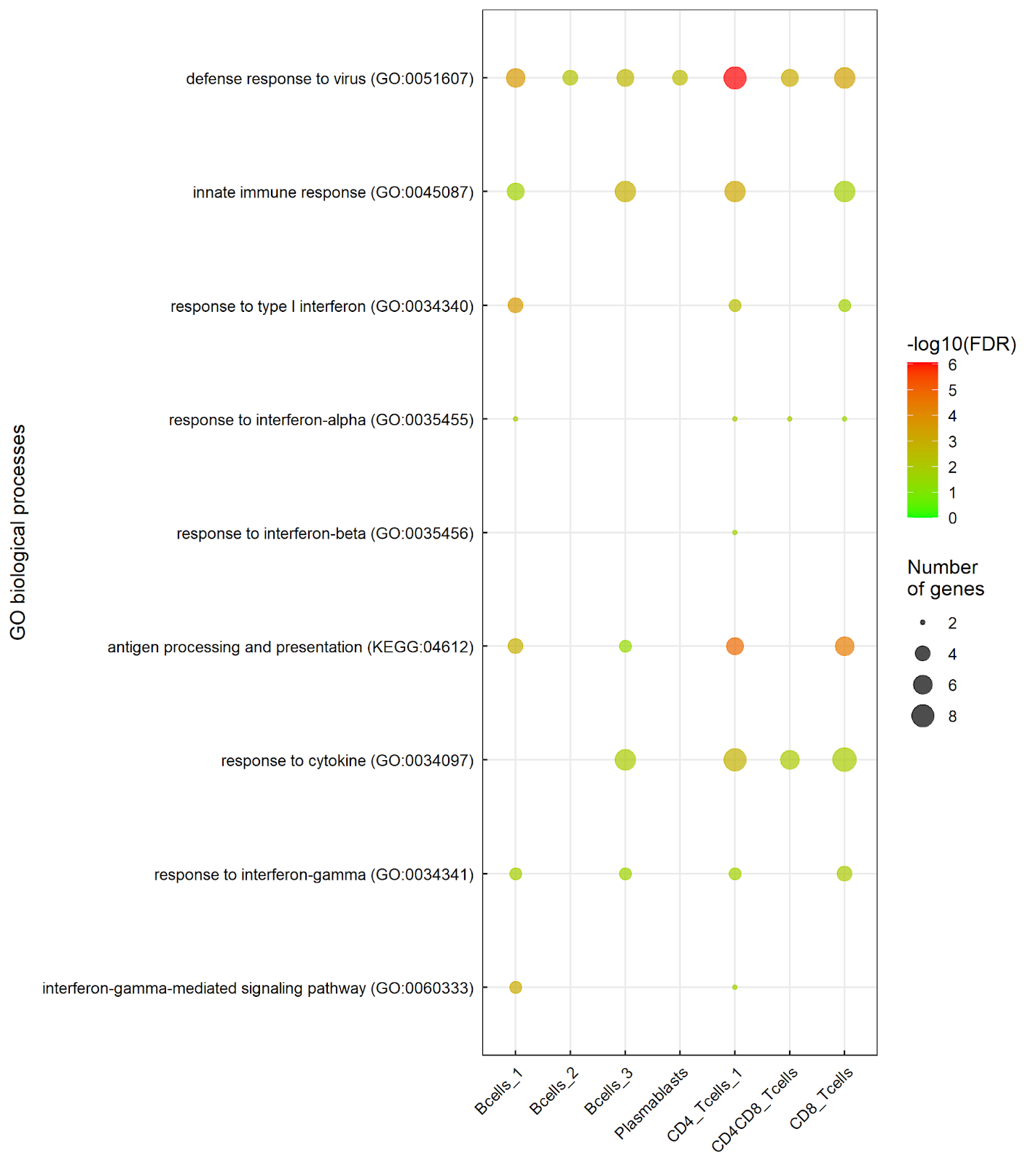

Supplement: S7 Fig — List of representative GO terms enriched for each cluster in DE genes from BA71ΔCD2-stimulated submandibular LN cells from the vaccinated pig. The size of the dots represents the number of DE genes associated with the GO term, and the color indicates the negative log10 value of the false discovery rate (FDR). (DOCX) [file ppat.1010931.s007.docx]

**S8 Fig.**


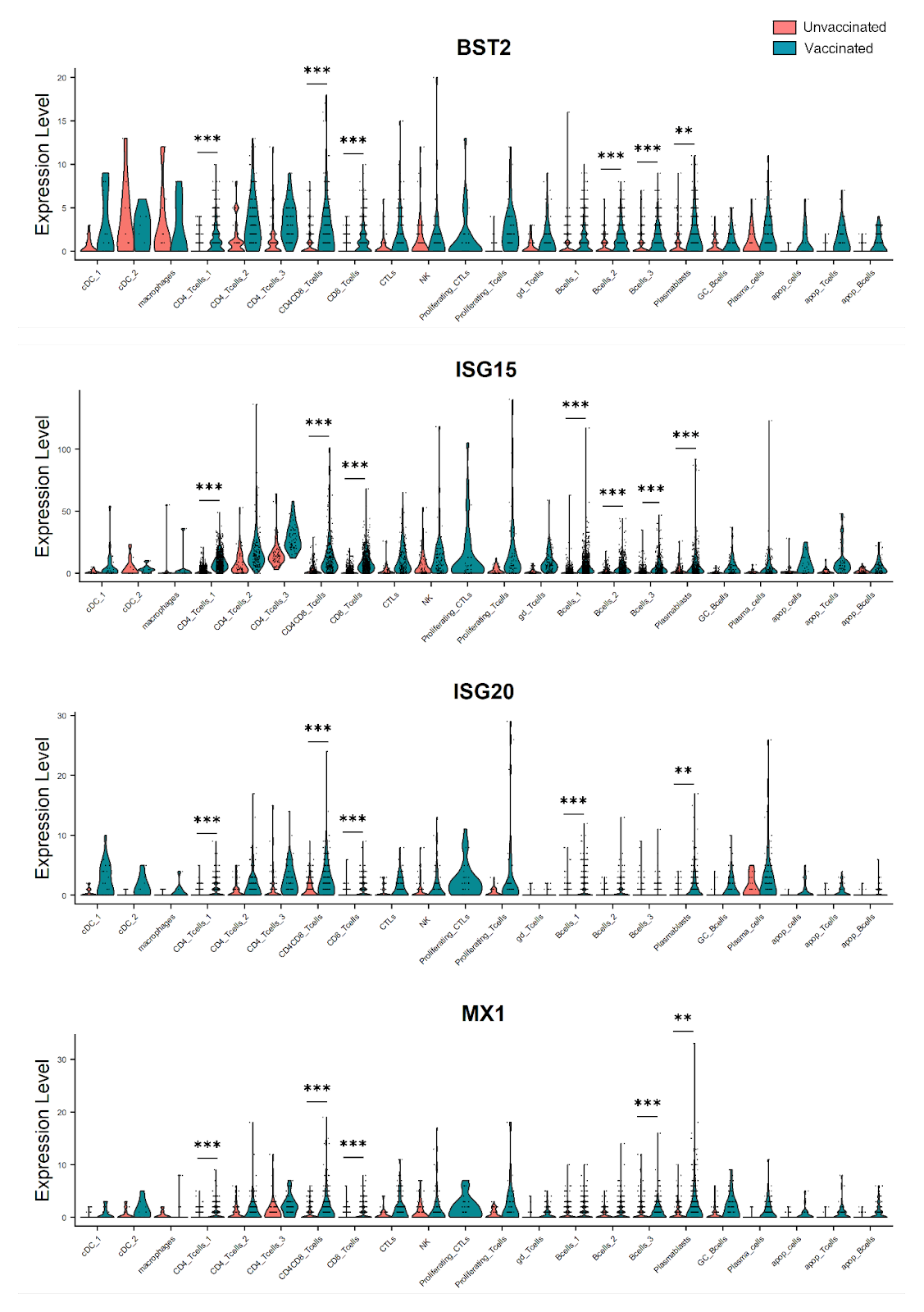

Supplement: S8 Fig — Asterisks denote differential expression: ** p value adjusted ≤ 0.01, *** p value adjusted ≤ 0.001. (DOCX) [file ppat.1010931.s008.docx]

**S9 Fig.**


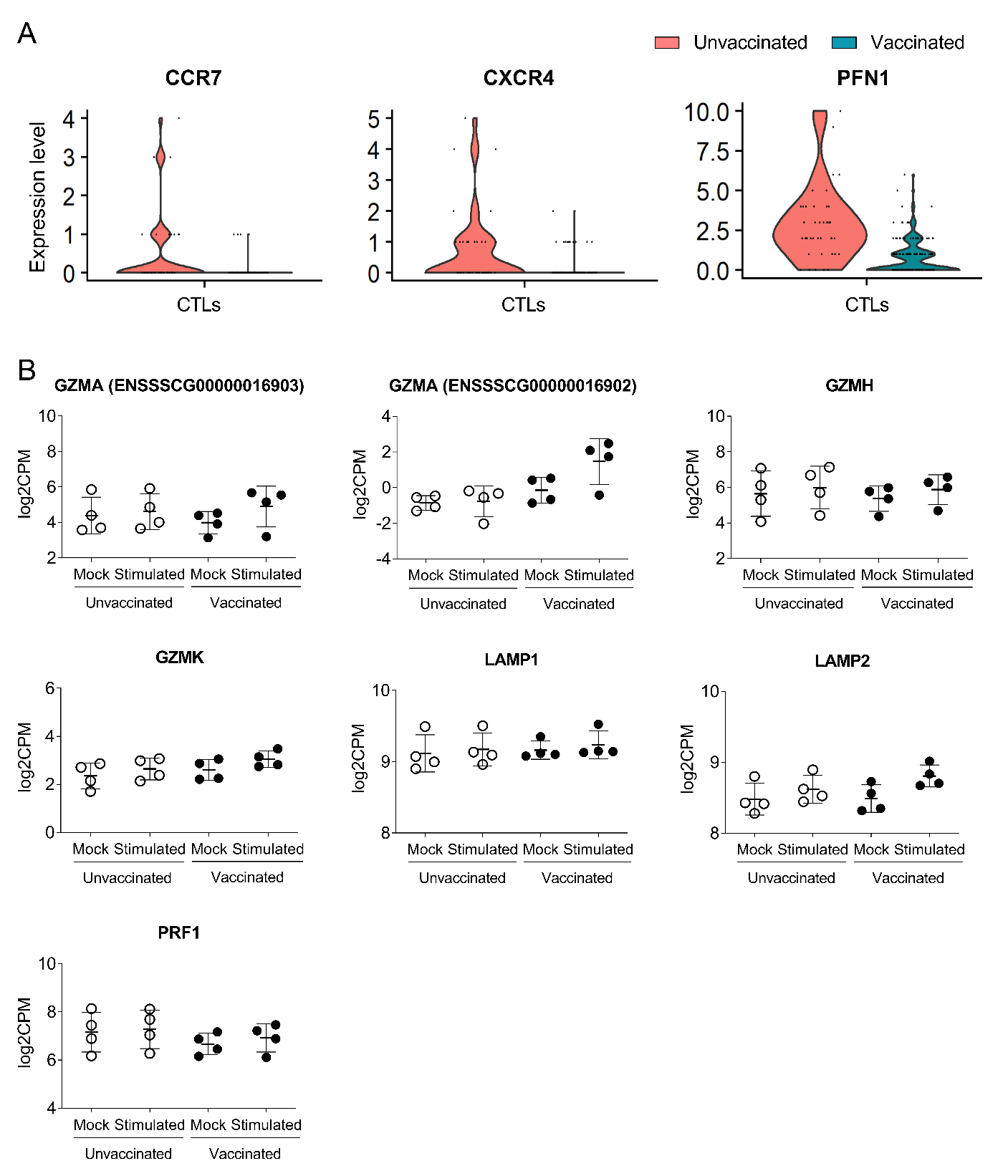

Supplement: S9 Fig — (A) Violin plots showing expression levels from scRNA-seq-derived data of CCR7, CXCR4 and profilin 1 (PFN1) in CTLs from LN cells after 16 hours of in vitro ASFV-specific stimulation. (B) RNA-seq-derived expression levels as log2CPM values of representative cytotoxic markers in PBMC after 10 hours of in vitro ASFV-specific stimulation. (DOCX) [file ppat.1010931.s009.docx]
