# Supplementary material for: Cross-protection against African swine fever virus upon intranasal vaccination is associated with an adaptive-innate immune crosstalk
Source: PLoS Pathog. 2022 Nov 9;18(11):e1010931. doi: 10.1371/journal.ppat.1010931 (PMC9645615; doi:10.1371/journal.ppat.1010931)
Supplement: S9 Table — (DOCX) [file ppat.1010931.s018.docx]

**Key resources table**

| REAGENT or RESOURCE | SOURCE | IDENTIFIER |
| --- | --- | --- |
| Antibodies | | |
| Peroxidase-conjugated rabbit anti-Pig IgG (whole molecule) | Sigma-Aldrich | Cat#A5670 |
| Purified mouse anti-pig IFNγ (Clone P2G10) | BD Biosciences | Cat#559961 |
| Biotin-labelled mouse anti-pig IFNγ (Clone 559958) | BD Biosciences | Cat#559958 |
| PE-conjuated mouse anti-pig IFNγ (Clone P2G10) | BD Biosciences | Cat#559812 |
| Pacific Blue-conjuated mouse anti-human TNFα (Clone MAb11) | BioLegend | Cat#502920 |
| FITC-conjugated anti-pig CD8α (Clone 76-2-11) | BD Biosciences | Cat#551303 |
| PerCP-Cy5.5-conjugated mouse anti-pig CD4α (Clone 74-12-4) | BD Biosciences | Cat#561474 |
| PE-Cy7-conjugated mouse anti-pig CD3ε (Clone BB23-8E6-8C8) | BD Biosciences | Cat#561477 |
| APC-conjugated rat anti-pig γδTCR (Clone MAC320) | BD Biosciences | Cat#561482 |
| FITC-conjugated mouse anti-pig CD14 (Clone MIL2) | Bio-Rad | Cat#MCA1218GA |
| PE-conjugated mouse anti-CD79a (Clone HM47) | eBioscience | Cat#12-0792-42 |
| eFluor450-conjugated mouse anti-human/pig perforin (Clone dG9) | eBioscience | Cat#48-9994-42 |
| Mouse anti-CD172a (IgG1, Clone BA1C11) | Provided by Dr. J. Domínguez | N/A |
| Mouse anti-SLAII/DR (IgG2b, Clone 1F12) | Provided by Dr. J. Domínguez | N/A |
| Mouse anti-CD163 (IgG1, Clone:2H12/BM) | Provided by Dr. J. Domínguez | N/A |
| APC-conjugated AffiniPure Goat Anti-Mouse IgG1 | Jackson ImmunoResearch | Cat#115-135-205 |
| Cy3-conjugated AffiniPure Goat Anti-Mouse IgG2a | Jackson ImmunoResearch | Cat#115-165-207 |
| Bacterial and virus strains | | |
|  |  |  |
|  |  |  |
|  |  |  |
|  |  |  |
|  |  |  |
| Biological samples |  |  |
|  |  |  |
|  |  |  |
|  |  |  |
|  |  |  |
|  |  |  |
| Chemicals, peptides, and recombinant proteins | | |
| 3,3′,5,5′-tetramethylbenzidine (TMB) | Sigma-Aldrich | Cat#T0440 |
| 3,3′,5,5′-tetramethylbenzidine (TMB), Insoluble | Sigma-Aldrich | Cat#613548 |
| Phorbol 12-myristate 13-acetate (PMA) | Sigma-Aldrich | Cat#P8139 |
| Ionomyicin | Sigma-Aldrich | Cat#I9657 |
| Phytohemagglutinin-M (PHA-M) | Sigma-Aldrich | Cat#11 082 132 001 |
| TRIzol™ Reagent | Invitrogen | Cat#15596026 |
| Chloroform | Sigma-Aldrich | Cat#372978 |
| Protein A, HRP conjugate | Millipore | Cat#18-160 |
| Acetate Buffer | Sigma-Aldrich | Cat#S7899 |
| N,N-Dimethylformamide | Sigma-Aldrich | Cat#227056 |
| Methanol | Merck | Cat#1.06018.2500 |
| Fetal Calf Serum | Corning | Cat#5535-016-CV |
| β-mercaptoethanol | Sigma-Aldrich | Cat#M3148 |
| Recombinant DNase I, RNase-free | Roche | Cat#4716728001 |
| Collagenase type IV | Gibco | Cat#17104019 |
| Critical commercial assays | | |
| Cytokine & Chemokine 9-Plex Porcine ProcartaPlex™ Panel 1 | Invitrogen | Cat#EPX090-60829-901 |
| Swine IFN gamma Do-It-Yourself ELISA | Kingfisher Biotech, Inc | Cat#DIY0725S-003 |
| LIVE/DEAD™ Fixable Violet Dead Cell Stain Kit | Invitrogen | Cat#L34955 |
| LIVE/DEAD™ Fixable Red Dead Cell Stain Kit | Invitrogen | Cat#L34972 |
| Fixation/Permeabilization Solution Kit with BD GolgiPlug | BD Biosciences | Cat#555028 |
| RNeasy Mini Kit | Qiagen | Cat#74106 |
| RNase-Free DNase Set | Qiagen | Cat#79254 |
| Agilent RNA 6000 Nano Kit | Agilent Technologies | Cat#5067-1511 |
| Prime Script RT reagent Kit | Takara | Cat#RR036A |
| Chromium Single Cell 3′ Library & Gel Bead Kit v3.1 | 10x Genomics | Cat#1000121 |
| Single Index Kit T Set A | 10x Genomics | Cat#1000213 |
| Deposited data | | |
| scRNA-seq data of porcine submandibular LN | This paper | GEO: GSE196472 |
| Bulk RNA-seq of porcine PBMC | This paper | GEO: GSE196473 |
|  |  |  |
|  |  |  |
|  |  |  |
| Experimental models: Cell lines | | |
|  |  |  |
|  |  |  |
|  |  |  |
|  |  |  |
|  |  |  |
| Experimental models: Organisms/strains | | |
| BA71ΔCD2 AFSV | IRTA, Spain | NA |
| Georgia2007/1 ASFV | Pirbright, UK | NA |
|  |  |  |
|  |  |  |
|  |  |  |
|  |  |  |
| Oligonucleotides | | |
|  |  |  |
|  |  |  |
|  |  |  |
|  |  |  |
|  |  |  |
| Recombinant DNA | | |
|  |  |  |
|  |  |  |
|  |  |  |
|  |  |  |
|  |  |  |
| Software and algorithms | | |
|  |  |  |
|  |  |  |
|  |  |  |
|  |  |  |
|  |  |  |
| Other | | |
|  |  |  |
|  |  |  |
|  |  |  |
|  |  |  |
|  |  |  |
