# Supplementary material for: Cross-protection against African swine fever virus upon intranasal vaccination is associated with an adaptive-innate immune crosstalk
Source: PLoS Pathog. 2022 Nov 9;18(11):e1010931. doi: 10.1371/journal.ppat.1010931 (PMC9645615; doi:10.1371/journal.ppat.1010931)
Supplement: Attachment — Submitted filename: Response to reviewers.docx [file ppat.1010931.s019.docx]

**Response to reviewers**

We would like to thank the reviewers for their thoughtful and thorough revision of our work. Their suggestions have been helpful to improve the manuscript.

We have now addressed the issues brought up, performed new experiments, and modified the manuscript as recommended. We hope you will find these changes and our response to the reviewers’ comments satisfactory.

Attached below you will find a point-by-point response to each of the reviewers’ comments. For clarity, we have highlighted the text written by the reviewers in black, and our response in blue. The key points of our response (including the changes brought to the figures and to the text) are written in **bold**.

**Reviewer #1**

**Summary:**

Bosch-Camos et al present here an analysis of the local and systemic immune response triggered by intranasal vaccination with attenuated African swine fever virus (ASFV). ASFV is a very important virus, expanding in a current zoonosis with a strong impact worldly on the porcine industry. Herein, the authors carried out four independent in vivo experiments on pigs and used up to date technics such as RNAseq and single cell RNAseq technics, backed up by flow cytometry analysis, allowing an accurate and robust description of the immune cells involved upon ASFV protective immune setting new references for further studies. Stand against these strong premises, it is a pity that the authors don’t go a little deeper on one of their main findings, the importance of myeloid cells activation or training in the efficacy of the BA71ΔCD2. In conclusion, although this work can be improved by deepening some investigations, it deserves publication in Plos Pathogens.

We thank the reviewer for the positive and constructive review and for considering that our work deserves publication in PLOS Pathogens. We agree that the potential role of training immunity in the vaccine-induced protection deserves further investigation, especially focusing on alveolar macrophages, which is out of the scope in the present work. Anyhow, we have performed a new experiment in this direction to demonstrate that the inflammatory response observed during the recall response of PBMC from vaccinated pigs is specific from ASFV, thus indicating a lack of trained immunity in blood monocytes/macrophages (see below in the point-by-point responses).

**Major Issues:**

1- Line 180, line 403, the authors evoke the possibility of monocytes training. Trained immunity has been shown to be induced by attenuated/recombinant vaccines (for review Goodridge Nat Rev Immunol 2016). One of the best publication, close to the work presented here, is Yao Cell 2018. An analysis of the alveolar macrophages phenotype and function would be out of the scope here, however one experiment investigating the in vitro cytokinic responses of post-vaccination monocytes to some TLR ligands such as LPS, Poly IC, R848, would add some mechanistic as well as a very interesting opening for further studies, in this highly descriptive work. In all cases some sentences in the discussion on the possibility of an indirect monocyte training, as observed in Yao et al. would be helpful.

As mentioned above, we agree that the analysis of the role of vaccine-induced trained immunity in the protection afforded by live attenuated ASFV vaccines is critical and deserves further investigations. **We have now modified the text in the discussion section** to address this point in more detail, also adding the two relevant references suggested by the reviewer (**lines 419**). We agree that the experiment proposed by the reviewer would be informative in this regard. However, we were not able to perform the experiment due to the lack of the required amount of blood. Instead, **we have performed a new experiment stimulating PBMC with CSFV and PRRSV, two porcine virus infecting monocyte/macrophages**. While stimulation with the virulent CSFV Margarita strain induce an inflammatory response in CD14+CD163+ monocytes/macrophages from both BA71∆CD2 vaccinated and unvaccinated pigs, stimulation with the attenuated PRRSV ALL-183 strain did not trigger TNF production by these same cells. Importantly, this experiment allowed to validate the presence of TNF-producing monocytes/macrophages from vaccinated pigs upon stimulation with our ASFV vaccine strain. These results demonstrate that the inflammatory response observed during the recall response of cells from vaccinated pigs is specific for ASFV, thus suggesting a lack of trained immunity in blood monocytes/macrophages. The results have been added in the **new figure 4G,** and the **text has been modified accordingly (lines 275 and 419).**

2- In the same line, the authors might restimulate PBMC with overlapping peptides of AFSV main antigens in order to untangle the innate immune cells activation trigger by the whole virus, from the antigen specific response triggered by the T lymphocytes.

We agree that peptide-based stimulations would allow to better understand to what extent the innate immune response observed during the recall response is triggered by ASFV-specific T cell responses. However, ASFV genome encodes more than 150 proteins, and very few of them have been described as immunogenic. In our experience, in contrast to *in vitro* stimulations using infectious virus, pools of these few peptides/proteins would not activate vaccine-induced T cell responses at the required levels for this kind of analyses (Bosch et al. 2021; doi: 10.3390/vaccines9010029). Thus, a lack of innate immune responses observed upon peptides/proteins stimulation could be a consequence of the activation of very low levels of ASFV-specific T cells. In addition, as mentioned in the previous point**, we have now included new results showing the specific nature of the inflammatory response observed during the recall response (new figure 4G).**

3- Line 268 and Fig 4B, please depict a really quantitative figure (mean+/- SD?) and not just this figure leading to a fuzzy visual feeling.

**We have modified Figure 4B**, which now shows levels of early and late ASFV transcripts grouped in a boxplot. In addition, **we have added in the text (lines 262 and 730)** the results of a Fisher test to quantitatively demonstrate the indistinguishable expression levels of viral transcripts in both animal groups.

4- Line 279: Figure 4F raised a very important point, as stressed by the authors in the discussion. However, no significant marks are depicted. If these data are not significant, either try to plot the % of reduction of TNF producing cells to get significant differences or make new experiments. If this is not possible, please don’t claim that ‘TNF production was abolished by blocking of IFNg’ (Line 279), and use more caution line 405 when stating ‘the dramatic reduction of TNF-producing macrophages when blocking IFNg’.

We agree with the reviewer that the results showed in Figure 4F were not conclusive due to the low number of samples analyzed. **We have repeated the experiment with new samples and performed the required statistical analysis to confirm the results**. In addition, **we have characterized the phenotype of the myeloid cells involved in TNF production**. The corresponding results are shown in the **new Figure 4F** showing the fold-decrease of TNF-production by CD14lowCD163+ myeloid cells upon IFNg blockage. **The text has been modified accordingly** **(lines 274 and 298)**.

5- Line 348: It is quite disturbing to validate LN scRNAseq using FACS analysis of PBMC. Please justify.

We apologize for the lack of clarity. Indeed, the term “validate” that we used in the manuscript was not appropriate. Importantly, our main aim was not to validate RNAseq results, but to demonstrate the induction of a systemic vaccine-specific cytotoxic response. Moreover, the obtention of fresh lymph nodes from vaccinated pigs before challenge with the virulent virus is difficult due to the experimental limitations when working with large animals in NBS3 facilities. In addition, the viability and responsiveness of frozen porcine immune cells is highly reduced during functional assays, in particular when stimulated with a cytopathic virus such as ASFV. For these reasons, we had to use the more accessible fresh blood samples to confirm the induction of cytotoxic cells in vaccinated pigs as revealed by scRNAseq of submandibular lymph node cells. Moreover, the fact that IFNg ELISpot assays showed similar numbers of ASFV-specific cells in PBMC and submandibular lymph node cells from vaccinated pigs (Figures 1C and S4A) suggested that the cytotoxic response might be also present in both tissues. **The text has been modified to clarify this issue (line 360).**

**Minor Issues:**

6- Figures’ legends are included in the main text. We included figure captions within the text after their first mention, following PLOS Pathogens guidelines.

7- In the abstract and/or author summary, it would be good to know a little more on the possibility to use this vaccine in the field, please evoke the biosafety. We have now included information in the mansuscript to address this issue (**line 505**).

8- Can you add some sentences on the mechanisms involved in the attenuation through CD2v depletion? For instance, do the attenuated virus still infect monocytes? Attenuation of BA71ΔCD2 is mainly based on its incapacity to bind to red blood cells, a CD2v-mediated mechanism that the virulent virus uses to rapidly spread through the body (Rodríguez et al., 1993; doi:10.1128/JVI.67.9.5312-5320.1993). Additionally, the cytoplasmic tail of CD2v has immunomodulatory functions (Chaulagain et al., 2021; doi:10.3390/v13081480), fact that might also contribute to the attenuation of the CD2v-depleted virus. However, BA71ΔCD2 infects monocytes/macrophages with similar efficiency than the parental virulent virus Ba71 (Monteagudo et al. 2017; doi: 10.1128/jvi.01058-17). **We have now included this information in the text (line 83)**.

9- Line 100, please specify that the memory CD4 and CD8 expanding after in vitro restimulation came from infected/vaccinated animals. We have now clarified this issue in the text (**line 106**).

10- Line 146, Figure 1D, the authors might process to a principal component analysis including all the read out of the vaccination. Although we agree with the reviewer that both ways to process the data are valid, we consider that the data as presented now is easier to understand for the broader reader.

11- Line 177, the sentence is not clear: ELISpot result does not ‘suggest the presence of a low number of ASFC-specific memory T cells in PBMC’, it reveals and precisely measure this low number. We agree with the reviewer that the sentence is not clear. We have **modified the text to clarify this issue (line 188)**.

12- Line 183: Please specify how you assure yourself of the random selection? We apologize for the lack of clarity. **We have modified the text to clarify this issue (lines 193 and 653)**. Also specify here that you chose the highest dose of vaccine in the following experiments. **We have modified the text** to specify it (**line 184**)

13- Legend Fig2A and 2B, please specify: left unstimulated, right ASFV stimulated. **We have modified the text** to clarify it (**lines 221 and 223**).

**Reviewer #2**

**Summary:**

In this manuscript, the authors investigated the immune response of domestic pigs to an infection challenge with African swine fever virus following vaccination with the live genotype I attenuated deletion mutant BA71ΔCD2. Pigs inoculated with a high dose (10^6) of BA71ΔCD2 survived a challenge when placed in contact with animals infected with the virulent heterologous genotype II strain Georgia 2007. Pigs that received lower doses (10^4.5 and 10^3) showed moderate to little protection against challenge in a dose-dependent manner, whereas the unvaccinated group succumbed 11-13 days post contact. Protection appeared to correlate somewhat with ASFV-specific antibodies in serum and number of IFNgamma+ PBMCs prior to contact challenge. Bulk RNAseq analysis of PBMCs of vaccinated animals revealed that restimulation induced strong innate and adaptive transcript profiles upon re-stimulation in vitro. In a second vaccination study (single high dose), PBMCs were re-stimulated with a homologous and heterologous strain in vitro 3 weeks after vaccination and a subset of transcripts were re-tested by digital PCR. Finally, submandibular lymph nodes from one vaccinated and one control pig were subjected to single cell RNA-seq 3 weeks after nasal vaccination revealing specific changes in lymph node cell subsets and associated transcriptional responses. The study provides new insights that will be informative for understanding correlates of protection that may help in rational design of vaccines against ASFV, arguably the pig pathogen of the greatest importance for animal health. The manuscript is well written, and the data accurately presented.

We thank the reviewer for his/her positive feedback and the suggestions made. We believe that with her/his help the manuscript gains in quality and clarity.

**Major Issues:**

1- One caveat to be discussed is that the vaccinated pigs are challenged only 3 weeks after vaccination. The vaccine virus is still detectable in serum and swabs of vaccinated animal (S2A/B Fig, T=0). It is likely that more virus will be found in whole blood (usually 2-3 logs higher than in serum) and potentially also in the submandibular lymph nodes. What is the impact of the presence of the virus on the innate response during in vitro recall and in vivo challenge?

We thank the reviewer for bringing this important issue up. Indeed, virus levels quantified by qPCR might significantly differ in whole blood compared to serum with most ASFV strains. However, this is largely due to the binding of ASFV to red blood cells through the CD2v protein, which our vaccine prototype BA71ΔCD2 lacks. Thus, BA71ΔCD2 levels detected by qPCR in serum are very similar to those in whole blood. To demonstrate it, we have performed the corresponding qPCR in samples from days 0, 14 and 21 post-vaccination. **The results are shown in the new table S1, and the text has been modified accordingly (line 135).** These results, together with the lack of viral transcripts in mock-stimulated PBMC as shown in Figure 4B, indicates that the recall response analyzed in PBMC by RNA-seq is not influenced by the possible presence of remaining virus. Moreover, and linked to the point 3 discussed below, **we detected low levels of viral DNA in submandibular lymph node cells after 16 hours of *in vitro* culture** and only in 2 out of the 6 samples from BA71ΔCD2-vaccinated animals **(new table S6)**. Anyhow, this does might not imply the presence of viable virus. Altogether, the results indicate that the remaining DNA (or virus) found in some samples from vaccinated pigs does not influence the *in vitro* recall response analyzed. To clarify this issue, **we have modified the text (line 320).**

2- Did the author try a genotype-specific qPCR to detect any sign of replication of the vaccine strain upon challenge with Georgia 2007 in the first experiment?

As suggested by the reviewer, **we have now performed a qPCR targeting the gene LacI, which is specifically contained in the genome of the BA71ΔCD2 vaccine virus**. We tested whole blood samples from vaccinated pigs harvested at days 10 and 13 post-challenge with Gerogia2007/1. All samples showed negative results, demonstrating the lack of BA71ΔCD2 replication. Samples from nasal swabs at days 7 and 14 post-vaccination, as well as whole blood from a naïve pig containing BA71ΔCD2, were used as technical positive controls. **The results are shown in the new table S2, and the text has been modified accordingly (line 147 and 602).**

3- What is the status of viremia and virus load in the lymph node in the second experiment at the time of recall and RNA seq. Was it tested?

As above mentioned, and answering the reviewer request, **we have now performed a qPCR to quantify viral DNA in submandibular lymph node cells after 16 hours of *in vitro* culture (new table S6)**. As mentioned above (see point 1), we detected low levels of viral DNA only in 2 out of 6 vaccinated animals, and the two samples used for scRNA-seq (marked in the table with an asterisk) were negative. **We have now added this information in the text** **(line 320).**

4- Did the authors record clinical signs and temperature after vaccination? It should be shown in supplementary materials for both experiments if available.

We have added this information in the **new supplementary figures S1 and S5, and modified the text accordingly (line 134 and 317).**

5- The adaptive-immune crosstalk link is somewhat speculative (line 387-389; 404-406). Blockade of IFNg blocked TNF expression but the mechanistic (“dependence”) link to CXCL10-mediated inflammatory response was not shown, only association can be deduced from RNAseq data.

We agree with the reviewer and have **changed the text accordingly (line 418).**

6 and 7- The lack of IFNg detection by ELISA is puzzling in Figure 1E given the ELISPOT and transcriptomic results. Can the authors exclude a technical issue with the multiplex ELISA using a positive control stimulation of naïve PBMCs? (we had issues with very low bead IFNgamma bead counts with the same kit). On the same point, the proposed justification as to why there is no IFNgamma in supernatants “the presence of a low number of ASFV-specific memory T cells in PBMC” (lines 178-179) does not make sense. The authors also contradict this point lines 196/197.

We thank the reviewer for bringing this issue up. However, **we have now quantified IFNG in supernatants by ELISA (Kingfisher Biotech, Inc.)**, and the results have confirmed the undetectable levels of IFNG in these samples. We hypothesize that the IFNG detected by ELISpot and transcriptomic analyses (two highly sensitive techniques) and produced by a low number of virus-specific T cells, is diluted in supernatants below the limit of detection of the ELISA and multiplex Luminex assay. To clarify this issue, **we have modified the text (lines 187 and 635**). However, if the reviewer considers it for the sake of clarity, we could eliminate the data showing the lack of IFNG in supernatants from the figure 1E.

8- In figure 4E, the specificity of the myeloid response in producing TNF is not clearly demonstrated. It would be better shown by adding a non-specific stimulation of PBMC with the heterologous ASFV Georgia 2007 virus or better with another type of virus infecting porcine macrophages. This may help reveal whether the myeloid response is truly specific or whether there is just non-specific innate priming (e.g. trained immunity response, epigenetic changes) that would not be as long lasting. The blockade of IFNg, while elegant and informative, is not sufficient to rule out an innate source of the cytokine (e.g., NK cells).

We agree with the reviewer that TNF-producing myeloid cells might be also primed as a result of vaccine-induced trained immunity. To address this issue, and as requested by the reviewer, **we have performed a new experiment using frozen PBMC from vaccinated and unvaccinated pigs**. Cells were stimulated for 8 hours with two porcine virus infecting monocytes/macrophages: the attenuated PRRSV ALL-183 strain, or the virulent CSFV Margarita strain. **The resulting percentages of TNF-producing CD14+CD163+ monocytes/macrophages are shown in the new Figure 4G**. The results demonstrate that the induction TNF-producing myeloid cells occurs only in PBMC from BA71ΔCD2-vaccinated pigs upon specific stimulation with the vaccine ASFV strain. We have included the results in the manuscript (**line 275 and 302**), and further discussed them in (**line 419**).

**Minor Issues:**

9- The setup of the first experiment needs to be clarified. Line129, the group of unvaccinated animals is not initially presented. Is it distinct from the challenged contact animals?

We apologize for the lack of clarity. **We have modified the text (line 133)** to specify that the group of unvaccinated pigs is different from the challenged contact animals. **We have also detailed further the *in vivo* experimental set up in M&M (line** **557)**.

10- Methods indicate that a ratio of 2:1 of vaccinated to intramuscularly injected pigs. However, it is not clear if all animals in the 4 groups (3 vaccinated and control) and the contact infected animals were all in a single large stable/room or if there were subgroups. If there were subgroups, please describe the setup. The heterogeneity of the disease course in the 2 groups receiving low dose of vaccines may be random or caused the variability of viral shedding by the contact intramuscularly infected pigs.

**We have modified the text in M&M** to describe the experimental set up in more detail (**line 557**). Briefly, the three groups of vaccinated pigs were housed in three independent experimental boxes, and each of them also containing two unvaccinated control pigs (making a total of 6 control pigs). Importantly, as shown in Figure 1B and S3A and B, all unvaccinated control pigs succumbed to the direct-contact challenge showing high virus loads in sera and nasal cavities.

11- What does “GP style” mean in figure legends? We apologize for the lack of clarity. “GP style” means GraphPad style (p > 0.05 ns, * p ≤ 0.05, ** p ≤ 0.01, *** p ≤ 0.001, **** p ≤ 0.0001). We have now erased the term “GP” from the manuscript and instead named it with the complete name (GraphPad).

12- Figures 2 and 3. XCL1 is a chemokine. It is grouped with cytokines. We apologize for the mistake, which we have fixed in the two figures.

13- S2 Fig. y axis labels and titles are missing. We apologize for the mistake, which we have now fixed.

14- The use of cDC_1 and cDC_2 as a labels for the subsets of cross-presenting DCs is not judicious as this may be confused with usual acronym for conventional dendritic cells (cDC1 and cDC2). Perhaps xDC may be a better choice to avoid confusing readers. We agree with the reviewer, and we have modified the labels as requested.

15- Line 342, spell out profilin 1 (to avoid mix up with perforin). We have changed the text (line 355) and the corresponding figure legend as requested.

16- Line 351: “significantly” should be used only in relation to statistical analysis. We apologize for the lack of clarity. The differences between stimulated and unstimulated cells were statistically significant, but we do not show these results in the figure (to improve clarity of the results, the values from unstimulated cells are subtracted from the stimulated ones). For this reason, we have deleted the term “significantly” from the text.

17- L384, the claim of priority (“first”) is perhaps unnecessary. We have deleted the term “first” from the sentence.

18- Fig 5A and 6A, the colors of some subsets are hard to decipher. The paucity of gd T cells is surprising. Have the authors confirmed presence T cell receptor alpha or beta chains in other T cell subsets? We agree with the reviewer that the number of gd T cells is lower than expected. The cluster was identified as gd T cells since they expressed CD3E, lacked CD2, and some of them expressed TRDC (T-cell receptor delta constant). Unfortunately, expression levels of T cell receptors alpha and beta were not high enough to be used to discriminate among different T cell subsets, probably due to the methanol fixation used in the protocol which resulted in a loss of quality of the data obtained. Moreover, we have observed by flow cytometry after *in vitro* stimulation a loss of many gd T cells as well as the downregulation of the gd T cell marker SWC6, that might explain the low number of gd T cells identified by scRNAseq.
